# CONNECTING THE HEALTH CARE WORKFORCE THROUGH A VIRTUAL LEARNING COMMUNITY

**DOI:** 10.1093/geroni/igad104.2125

**Published:** 2023-12-21

**Authors:** Judy Howe, Eve Gottesman, Eugenia Dorisca, Yuka Shichijo, Fred Ko

**Affiliations:** Icahn School of Medicine at Mount Sinai, New York City, New York, United States; US Department of Veterans Affairs/Zicklin School of Business, New York, New York, United States; James J. Peters VA Medical Center, Bronx, New York, United States; Icahn School of Medicine at Mount Sinai, New York City, New York, United States; James J. Peters VA Medical Center, Bronx, New York, United States

## Abstract

The COVID-19 pandemic highlighted the need for online and virtual educational opportunities and resources. While many healthcare organizations, hospitals and academic medical institutions were forced to quickly pivot from face-to-face training modalities, the Virtual Learning Community (VLC) of the Geriatric Scholars Program, was ahead of the curve. Established in 2014 by the VA Geriatric Scholars Program and the VHA’s Office of Rural Health, the VLC is the primary outlet for information on geriatrics and gerontology for healthcare professionals and staff. The VLC provides webinars, discussion forums, case studies, continuing education accredited programs, and resource materials on a myriad of topics related to aging. During the pandemic, the VLC provided up-to-date information on COVID and continued to augment its virtual offerings, which was vital to providers and staff working remotely. The VLC currently has approximately 5,000 registered users, with 1700 users registering since the pandemic began in March 2020. Many of these users come from rural and highly rural areas, where there is often a lack of geriatric knowledge and expertise. The presentation will present findings about the connections and impact made possible by a pre-existing web-based virtual learning community.

